# Ab Externo Imaging of Human Episcleral Vessels Using Fiberoptic Confocal Laser Endomicroscopy

**DOI:** 10.18502/jovr.v14i3.4783

**Published:** 2019-07-18

**Authors:** Ken Y. Lin, Sameh Mosaed

**Affiliations:** Gavin Herbert Eye Institute, Department of Ophthalmology, University of California, Irvine, USA

**Keywords:** Aqueous Outflow, Laser Imaging, Minimally Invasive Glaucoma Surgery

## Abstract

**Purpose:**

There is a growing interest in targeting minimally invasive surgery devices to the aqueous outflow system to optimize treatment outcomes. However, methods to visualize functioning, large-caliber aqueous and episcleral veins *in-vivo* are lacking. This pilot study establishes an *ex-vivo* system to evaluate the use of a confocal laser microendoscope to noninvasively image episcleral vessels and quantify regional flow variation along the limbal circumference.

**Methods:**

A fiber-optic confocal laser endomicroscopy (CLE) system with lateral and axial resolution of 3.5 μm and 15 μm, respectively, was used on three porcine and four human eyes. Diluted fluorescein (0.04%) was injected into eyes kept under constant infusion. The microprobe was applied to the sclera 1 mm behind the limbus to acquire real-time video. Image acquisition was performed at 15-degree intervals along the limbal circumference to quantify regional flow variation in human eyes.

**Results:**

Vascular structures were visualized in whole human eyes without processing. Schlemm's canal was visualized only after a scleral flap was created. Fluorescent signal intensity and vessel diameter variation were observed along the limbal circumference, with the inferior quadrant having a statistically higher fluorescein signal compared to the other quadrants in human eyes (P < 0.05).

**Conclusion:**

This study demonstrates for the first time that the fiber-optic CLE platform can visualize the episcleral vasculature with high resolution *ex-vivo* with minimal tissue manipulation. Intravascular signal intensities and vessel diameters were acquired in real-time; such information can help select target areas for minimally invasive glaucoma surgery (MIGS) to achieve greater intraocular pressure reduction.

##  INTRODUCTION

The last several years have witnessed a rapidly rising interest in both developing new and modifying current minimally invasive glaucoma surgery (MIGS) methods. This surge in momentum is spurred by the fact that the current standard glaucoma surgery methods, trabeculectomy, and tube shunt surgery, still have high complication rates, ranging from 27% in tube shunts to 74% in trabeculectomy.^[1,2]^ MIGS devices improve the outflow by bypassing or ablating the trabecular meshwork (TM) or creating new drainage routes into the suprachoroidal space.^[3]^ Clinical studies so far show that most MIGS procedures display improved safety profiles over traditional procedures. For instance, the most common complications are temporary hyphema and transient rise in intraocular pressure (IOP) in the early postoperative period, occurring in 3–10% of Trabectome^[4]^ and 2% of iStent patients.^[5,6]^

IOP reduction in MIGS is generally not as dramatic as those in trabeculectomies and tube shunts. Clinical studies show an average IOP of 15.2 mmHg for phaco-trabectome at five years and 16.8 mmHg for the iStent with phacoemulsification at five years.^[4][5,6]^ Since ablative type MIGS such as Trabectome and Kahook dual blade are capable of removing a large arc of TM, they can generally achieve lower IOP endpoints compared to the bypass type, such as iStent.^[3]^ However, this comes at an expense of higher rates of transient postoperative IOP rise and hyphema. Furthermore, anatomic studies have implied that Schlemm's canal is highly segmented and discontinuous, and the location and number of collector channels may vary among individuals.^[7,8]^ Bypassing an area of TM at great distance from the collector channels may thus not achieve the full potential of IOP reduction. These lines of evidence all suggest that the real estate value of the TM is not the same across the entire 12 clock hours. In other words, knowing what segment of the TM to ablate or bypass may be crucial for achieving more targeted MIGS and potentially narrow the gap between the IOP endpoints currently seen in MIGS and the theoretical limit of episcleral venous pressure.^[9,10,11]^

One intuitive approach to target the high-yield TM is to identify the collector channels via imaging.^[9]^ Several studies have successfully imaged outflow anatomy using 3D micro-computed tomography,^[12]^ swept-source optical coherence tomography (ss-OCT),^[13]^ and endoscopic OCT.^[14]^ However, all these techniques require extensive tissue processing, which limits clinical applicability.

Here, we tested the hypothesis that a commercially available fiber-optic confocal laser microendoscope can visualize episcleral vessels and characterize their density and diameters after intracameral fluorescein injection in cadaveric human eyes with minimal tissue manipulation. The microendoscope is the world's smallest microscope. It enables clinicians to perform real-time optical biopsy by visualizing the microvasculature in tissues as well as detecting abnormal cells. So far, it has been FDA-approved for use in the detection of gastrointestinal, urological, and pulmonary pathologies, from Barrett's esophagus and colorectal lesions to lung nodules and urinary track lesions.^[15,16,17,18]^

The first goal of the present study was to establish an *ex-vivo* system with cadaveric human eyes to determine if the microendoscope is capable of detecting episcleral vessels. The second goal was to determine if regional differences in episcleral vessel morphology can be detected and quantified across the 12 clock hours of the limbus. This pilot study aimed to pave the way for future studies to identify, preoperatively and noninvasively, areas of TM that MIGS procedures should access to yield the lowest postoperative IOP possible.

##  METHODS

###  Fiber-optic Confocal Laser Microendoscope

**Figure 1:**
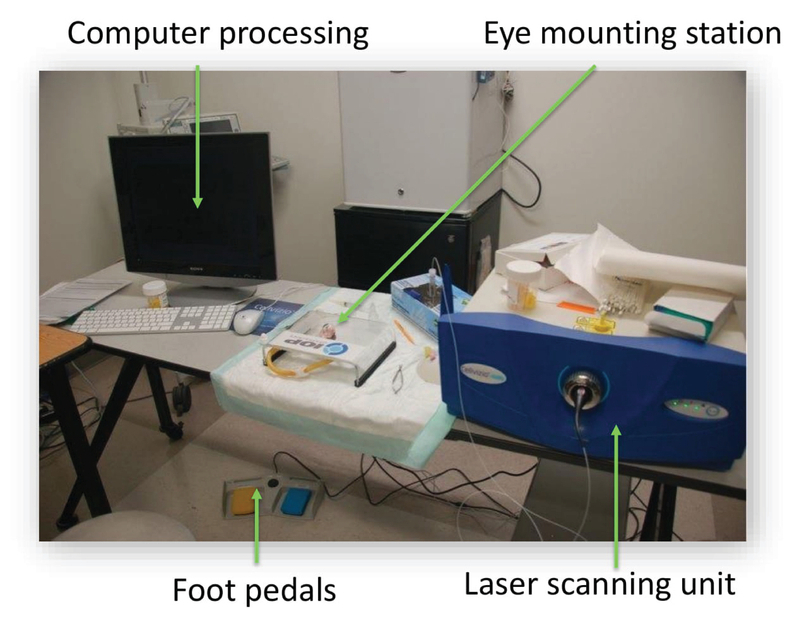
The fiber-optic confocal laser microendoscope imaging system. The laser scanning unit emits light at 488 nm, which is fed into the flexible fiber-optic core. The fluorescent emission is collected in the same fiber that was used for illumination. The microendoscope has a diameter of 1.5 mm and projects a field of view of 423 × 423 μm. Lateral and axial resolutions are 3.5 μm and 15 μm, respectively. The operator maneuvers the tip of the fiber-optic microendoscope to image different areas. Real-time image and video capture are activated via a foot pedal.

The imaging system, Cellvizio (Mauna Kea Technologies, Paris, France), has been described previously.^[15,19]^ Briefly, the system has three components [Figure 1]: a fiber-optic microendoscope, a laser scanning unit, and a computer. A 488-nm laser source is rastered by two mirrors on one end of a fiber bundle consisting of 30,000 optic fibers. The laser was sequentially directed into each fiber to reach the tissue. The fluorescent emission is collected in the same fiber that was used for excitation. The small core diameter of each fiber acts as a pinhole to give the probe optical sectioning capability. The microendoscope used in this study has a maximum depth of tissue penetration of 100 μm.

The microendoscope has a diameter of 1.5 mm and projects a field of view of 423 × 423 μm. Lateral and axial resolutions are 3.5 μm and 15 μm, respectively. This method had been previously compared to intravital fluorescent microscopy and histological staining in gastrointestinal tissues,^[15]^ and all methods yielded statistically similar measurements of luminal diameters in the microvasculature. A post-processing image frame or video is shown on a monitor to allow surgeons to navigate along the limbus in real-time.

###  Fluorescent Probe

Previous studies have injected fluorescein at 0.1–0.15% into the anterior chamber for purposes of fluoroscopy.^[20]^ Much lower concentrations of fluorescein are likely sufficient to produce contrast when imaged with a confocal microendoscope. Fluress${}^{\mathrm{TM}}$ contains 0.25% fluorescein and was diluted to 0.04% through serial 1:1 dilutions with balanced salt solution (BSS).

### *Ex-vivo* Perfused Porcine and Human Eye Models

**Figure 2:**
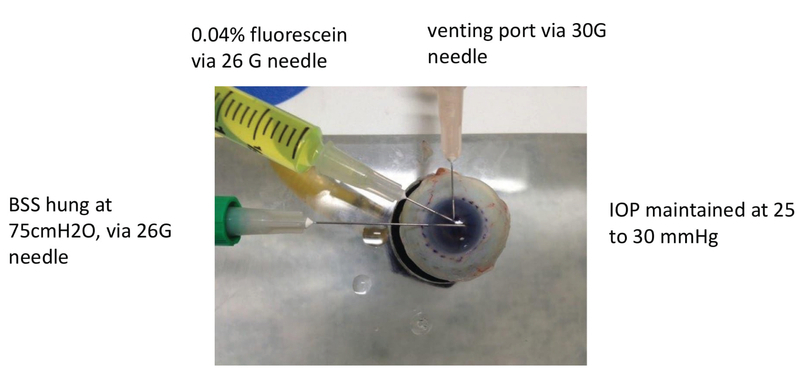
Eye perfusion system. Balanced salt solution (BSS) solution was delivered into the eye via a 26-gauge needle. Intraocular pressure (IOP) was maintained at between 25 and 30 mmHg. A smaller caliber 30-gauge needle was used to create a venting port at 90${}^{\text{o}}$ away from the infusion port. A 26-gauge needle was inserted half-way between the infusion and venting ports to inject fluorescein.

Three porcine eyes were used (Alcon, Irvine, California). The whole eye was first mounted onto a flat platform and secured with vacuum suction provided via a syringe [Figure 2]. The iris plane was placed parallel to the ground and confirmed with Axis Assistant, an iPhone-based application. A 360${}^{\text{o}}$ limbal peritomy was performed using mini-Westcott scissors, and the Tenon's membranes were bluntly dissected to expose the bare sclera. BSS solution was placed at a 75 cmH2O position and delivered into the eye via a 26-gauge needle. A smaller-caliber 30-gauge needle was used to create a venting port at 90${}^{\text{o}}$ away from the infusion port. We have found that the creation of a venting port helps maintain stable pressure throughout the infusion process, since supraphysiologic pressure may potentially cause Schlemm's canal to collapse. This bottle height and vent system were chosen as, together, they provide our *ex-vivo* perfused eye platform an IOP of 25–30 mmHg. The IOP was checked every three minutes by Tonopen. A 26-gauge needle was inserted half-way between the infusion and venting ports to inject 0.2 cm${}^{3}$ of 0.04% fluorescein over five seconds once the tip of the needle reached a point 1 mm above the center of the anterior lens capsule with a bevel-up position to minimize the preferential delivery of fluorescein to a particular quadrant of the angle.

Four human eyes from two individuals were obtained from SightLife Surgical (Irvine, Ca). The cadaveric human eyes were from a 78-year-old man and an 82-year old man, respectively, with no history of glaucoma or eye surgery. The eyes from the 82-year-old man were used for the initial study to determine the optimal image time window after fluorescein injection, while the eyes of the 78-year-old man were used for all subsequent studies. Each eye was mounted onto the perfusion platform as described earlier. The superior pole of the eye was identified based on the relative location of the optic nerve to the inferior oblique muscle insertion. Both human eyes had only trace amounts of conjunctival and Tenon's tissues and no dissection or tissue manipulation was performed on the human eyes prior to mounting them.

###  Scleral Flap Construction

To facilitate visualization of Schlemm's canal, which lies approximately 200 μm underneath the sclera deep beyond the microendoscope's 100 μm penetration depth, a triangular limbal scleral flap was created. Briefly, a no. 69 Beaver blade was used to create a 3 × 3 mm triangular scleral flap with its base at the limbus. The blade was used to create two incisions at 50–75% of the scleral depth beginning at the posterior end of the surgical limbus extending toward the apex of the triangle. Next, the no. 69 Beaver blade was used in an almost horizontal manner across the apex of the triangle to lift the edge of the flap from the bottom of the grooves. Non-toothed forceps were then used to lift the flap and create traction between the flap and scleral bed. The no. 69 blade was used in a horizontal position to cut across the scleral tissue. The dissection was carried anteriorly until the blue–gray zone of the limbus was visible. The microendoscope was then placed above the blue–gray zone to image Schlemm's canal. Image acquisition was initiated immediately after fluorescein injection into the anterior chamber.

**Figure 3:**
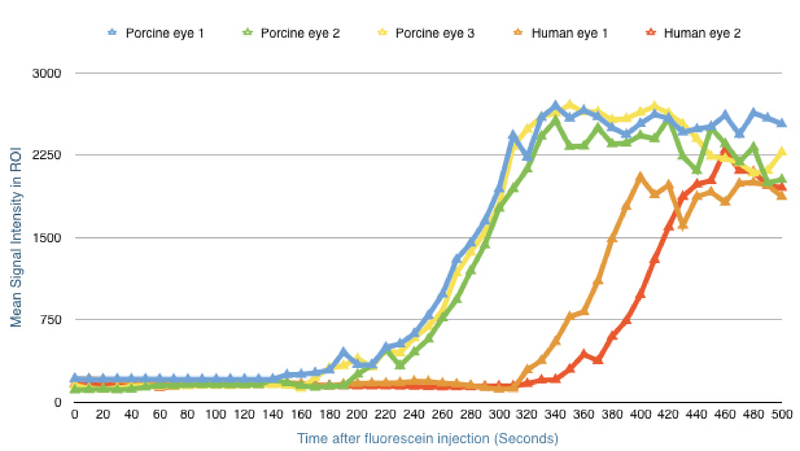
Rise in fluorescence after fluorescein injection into the anterior chamber in porcine and human eyes. The units are fluorescent arbitrary units (au). The plot shows that the optimal time to capture image is five minutes after injection in porcine eyes and seven minutes after injection in human eyes. ROI, region of interest

**Figure 4:**
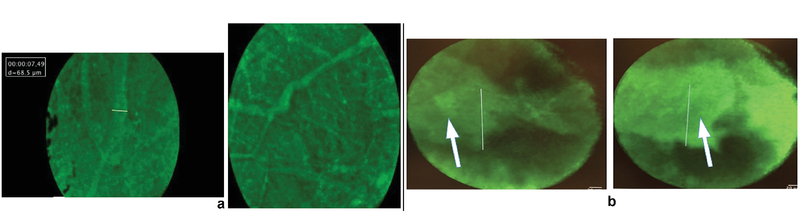
Visualization of episcleral vessels (a) and Schlemm's canal (b) with minimal tissue manipulation. Episcleral vessels are seen emanating from deeper, larger-caliber vascular structures. The white bar represents a length of 50 μm. Visualization of Schlemm's canal (white arrow) is only possible after a scleral flap is introduced because the microendoscope is limited to a depth of 100 micron in scleral tissue. The white bar in (b) represents a length of 120 μm.

**Figure 5:**
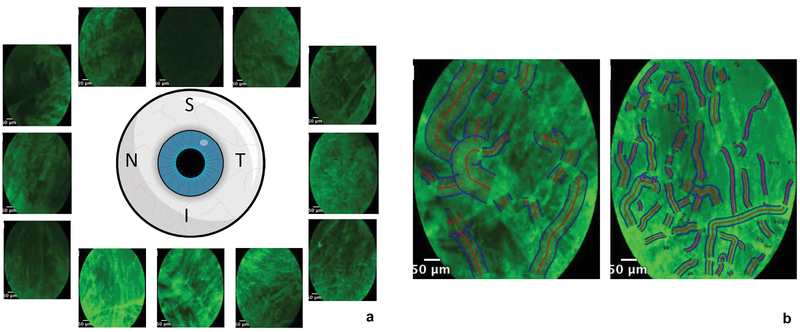
Distribution of fluorescence signal along the limbal perimeter. Representative frames from individual quadrants were chosen to highlight the differences in fluorescence signals along the limbus (a). The differences can be quantified using a vessel segmentation algorithm, which automatically detects the vessel border and midline (b) and quantifies vessel diameter and signal intensity in the chosen region of interest.

**Figure 6:**
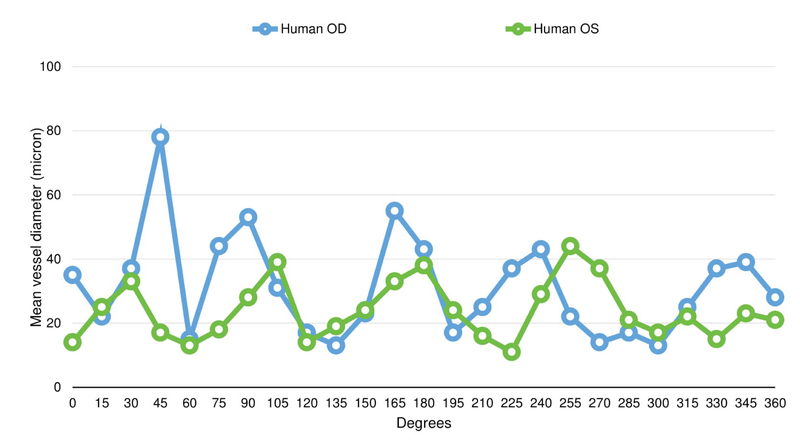
Episcleral vessel diameter distribution along the limbal perimeter in human eyes. Larger-diameter episcleral vessels appear to group in clusters that span 40–50${}^{\text{o}}$.

**Figure 7:**
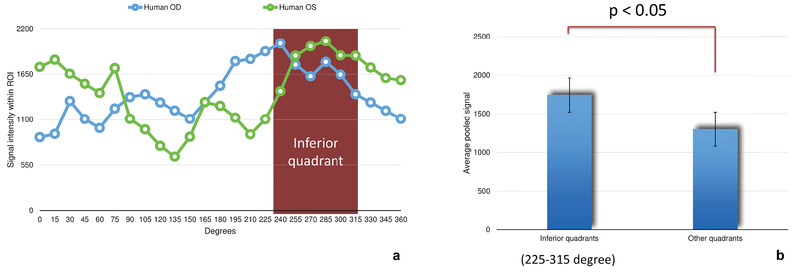
Fluorescent signal intensity distribution along the limbal perimeter in human eyes. (a) Signal intensity appears to concentrate more in the inferior quadrant in both human eyes. (b) The inferior quadrant has a statistically higher amount of fluorescence when compared to all other quadrants combined. Fluorescence was measured in arbitrary units (au). ROI, region of interest.

###  Determining Optimal Time for Imaging After Fluorescein Injection

The eye was mounted as previously described, and the microendoscope was gently pressed against the limbus at 180${}^{\text{o}}$ away from the infusion port. Video acquisition was initiated by the surgeon via a foot pedal after the completion of the fluorescein injection. The microendoscope was held stationary by the surgeon to maintain the same view and the increase in intraluminal fluorescence was observed in real-time on the monitor for eight minutes. The same protocol was used for both porcine and human eyes.

Image analysis was performed using ImageCell (Mauna Kea Technologies, Paris, France). A circular region of interest (ROI) was manually marked to match the field of view of the microendoscope. The total signal intensity within the ROI at each frame was calculated and plotted against time.

###  Episcleral Vessel Segmentation

To quantify vessel diameter and fluorescent signal along 360${}^{\text{o}}$ of the limbus, we first marked the limbus with a surgical blue marker placed at 15${}^{\text{o}}$ intervals [Figure 2]. Still images at each mark were acquired at five minutes for porcine eyes and seven minutes for human eyes. Fluorescent intensity was expressed as fluorescent arbitrary units (au).

A vessel segmentation algorithm was applied to each frame to outline the border of the lumen and compute the vessel diameter. This method had been previously described and validated.^[21,22]^ Briefly, vessels were modeled as tubular structures with the intraluminal signal intensity displaying a Gaussian distribution with the signal maxima denoting the luminal center, or “ridge.” Given that vessel diameter can have local variations along the direction of the lumen, the ridges were detected at different scales, the medium response was computed, and the maxima of the responses through the scales were recorded. The scale at which a maximum ridge was detected led to an estimate of vessel radius. The segmentation algorithm thus generated several sets of connected points, with each set representing a vessel midline and each point within a set carrying associated diameter information. These points were denoted and overlaid onto the original image to provide a graphical representation of where the vessel borders were. A data table containing information on vessel diameters could then be exported to Excel for statistical analysis.

###  Statistical Analysis

All values in this study are expressed as mean +/– standard error of the mean. No statistical analysis was performed on times of signal increase after fluorescein injection in three porcine and two human eyes as these were observational experiments aimed at determining the optimal timeframes for data collection in subsequent experiments. For comparison of signal intensities between the inferior quadrants vs. all other quadrants, the signals from the inferior quadrants, defined as the areas from 225${}^{\text{o}}$ to 315${}^{\text{o}}$ from two human eyes were compared with signals from all other quadrants. A two-tailed *t*-test was used to determine statistical significance, where a *P* < 0.05 denotes significance.

##  RESULTS

###  Optimal Time for Detection after Fluorescein Injection

Porcine eyes began to reach the plateau phase of fluorescent signal intensity five minutes after fluorescein injection. For human eyes, the peak time occurred at seven minutes after fluorescein injection [Figure 3].

###  Visualization of Episcleral Vessels and Schlemm's Canal

Episcleral vessels with diameters ranging from 10–50 μm were seen emanating into the scleral surface [Figure 4(a)]. The images were obtained from human eyes without tissue processing. The microendoscope did not visualize Schlemm's canal ab externo because its tissue penetration is less than 100 μm. Construction of a scleral flap can assist in visualizing Schlemm's canal, which was confirmed by the dimensions of the tubular structure (75–100 μm) and its peri-limbal location [Figure 4(b)]. However, we were able to maintain the view to Schlemm's canal for only fewer than 10 seconds after fluorescein injection, likely because, as the eye was actively perfused, fluorescein was egressing the intrascleral vessels along the cut edges of the scleral flap.

###  Differences in Vessel Morphology Along the Limbus in Human Eyes

Regional variation in fluorescein intensity along the limbus could be qualitatively appreciated [Figure 5(a)]. A vessel segmentation algorithm was applied to individual frames acquired at 15${}^{\text{o}}$ intervals along the limbus. An example of a composite image is shown where the vessel border is outlined in blue and the vessel midline is marked in red [Figure 5(b)].

The distribution of vessel diameters is non-uniform along the limbus in human eyes, with larger-diameter episcleral vessels seemingly grouped in clusters that span 40–50${}^{\text{o}}$ [Figure 6].

Fluorescent signal intensity appears to concentrate more in the inferior quadrant in both human eyes [Figure 7(a)]. In fact, when compared with the other quadrants combined, the inferior quadrant has a statistically higher amount of fluorescence (1742 +/– 271 au vs. 1300 +/– 316 au, *P* < 0.05) [Figure 7(b)].

##  DISCUSSION

The salient findings of this study are that: (1) a confocal laser microendoscope can visualize episcleral vessels in cadaveric human eyes with minimal tissue manipulation, (2) in our *ex-vivo* system involving a perfused eye, the optimal time to image episcleral vessels is seven minutes after fluorescein injection into the anterior chamber, and (3) episcleral vessel diameter and density can be acquired in real-time and show that the inferior quadrants of the two human eyes in this study have statistically greater amount of fluorescence compared to the other quadrants.

This study showed that the microendoscope was able to image surface episcleral vessels. The flexibility of the microendoscope catheter also enables the operator to easily rotate the endoscope to image other areas of the limbus. As discussed later, the ability to image episcleral vessels noninvasively may be of great diagnostic value.

One factor that potentially limits the extent of IOP reduction following MIGS is the lack of knowledge on which portion of the TM to ablate or bypass. The idea that location may play a crucial role in determining post-operative IOP has spurred much interest in noninvasive imaging of the conventional outflow pathway.^[9]^ One group has recently visualized the angle structure using swept source OCT;^[13]^ another group has visualized the intrascleral plexus using micro-CT. Micro-CT has revealed, in great detail, the complexity of intra- and episcleral vessels;^[12]^ however, this imaging modality is limited to *ex-vivo* settings.

While swept source OCT has great clinical translatability, the authors admitted that image acquisition of the entire 360${}^{\text{o}}$ may require up to several seconds and can be confounded by motion artifacts.^[13]^ In addition, studies in mice^[23]^ and humans^[8]^ have shown that Schlemm's canal can partially collapse under increased IOP. This can make consistent identification of the angle structures more challenging. Lastly, OCT provides ample anatomical data, but functional correlates to guide the choice of locations for MIGS are currently still lacking.

A contrast-enhanced technique may be needed to help highlight the lumen of the outflow pathway. An intraoperative technique has recently been proposed to locate larger-caliber aqueous veins by observing episcleral venous fluid waves.^[24]^ This technique is obviously limited to intraoperative use and is a largely qualitative and subjective method. However, it may be of value in cases where obvious sectoral differences in flow along the limbus exist. The confocal laser microendoscope in this study uses fluorescein to provide contrast. Because the intracameral fluorescein would follow and thereby outline the functioning aqueous veins and episcleral vessels, the diameter and density of the episcleral vessels serve as a surrogate functional marker for flow associated with the limbal area imaged by the microendoscope.

This pilot study shows that a vessel segmentation algorithm can quantify episcleral vessel morphology; furthermore, vessel diameter and the amount of flow both show regional variation, and that flow is more concentrated in the inferior quadrant in the two human eyes studied. Finally, the flexibility of the microendoscope and the foot pedal also enhance the operator's maneuverability. Both of these features augment the clinical applicability of the microendoscope, as image acquisition can be performed in an ambulatory outpatient setting.

There were two recent studies showing more collector channels in nasal and inferior quad- rants.^[25,26]^ This is not necessarily in disagreement with our observations in this study. The study by Li et al compares nasal vs. temporal quadrants, but does not compare other quadrants.^[25]^ As such, their findings were not intrinsically incompatible with our findings. In addition, our observations were based on one pair of eyes from the same individual, and greater numbers are needed to determine if this is generalizable to a larger population. Moreover, enhanced depth OCT in the study by Li et al nicely delineated the anatomy of the collector channel, but this may not imply that these open collector channels necessarily experience flow through them. There may be differences between contrast based imaging vs. anatomy based imaging, as the former visualizes actual flow and is thus more functional, while the latter reflects anatomy. The study by Cha and colleagues did show that nasal and inferior quadrants contain the most collector channels. In summary, we do not believe that our results are inherently incompatible with these published results due to (1) the fact that our study lacked enough statistical power to support such a statement and that (2) functional vs. anatomic imaging may lead to different conclusions, as one looks for open and functioning collector channels while the other looks at open collector channels—this is mostly hypothetical at this point and would be an interesting topic for a future study.

Although fluorescein had been shown to be safe for intraocular use in human eyes,^[20]^ future studies can potentially use indocyanine green to provide the source of contrast. A laser scanning unit that excites at the peak absorption of 600 nm of indocyanine is currently being developed to facilitate this goal. The current investigation is a feasibility study performed on a small number of cadaveric eyes. Another limitation of the study is that fluorescein extravasation restricts the time window of image acquisition in eyes *ex-vivo*. Additional studies are needed to establish if a greater amount of flow in the inferior quadrant, for instance, is specific to an individual or is generalizable to the population. Future animal studies are also needed to determine if similar signal to noise ratios and resolutions still hold in live eyes. Although not yet clinically applicable in its current form, contrast-enhanced confocal laser microendoscopy may be one viable method to image intra- and episcleral vessels and ultimately help surgeons target MIGS to areas of higher scleral flow. In other words, this is the first step toward the ultimate goal of clinical integration. Additional optical modifications are already underway to refine the method presented in this pilot study and to ultimately make it more clinically applicable.

##  Financial Support and Sponsorship

American Society of Cataract and Refractive Surgery (ASCRS) Foundation Grants, 2014.

##  Conflicts of Interest

There are no conflicts of interest.
